# A Prosthetically Coupled Tripod Fixation Concept for Edentulous Surgical Guides: A Three-Case Proof-of-Concept Study

**DOI:** 10.3390/dj14060385

**Published:** 2026-06-22

**Authors:** Ioan-Achim Borșanu, Ralph-Alexandru Erdelyi, Sergiu-Manuel Antonie, Remus Christian Bratu, Emanuel-Adrian Bratu

**Affiliations:** 1 Clinic of Implant Supported Restorations, “Victor Babeș” University of Medicine and Pharmacy Timisoara, 2 Eftimie Murgu Sq., 300041 Timisoara, Romania; antonie.sergiu@umft.ro (S.-M.A.); or remusbratuchristian@gmail.com (R.C.B.); ebratu@umft.ro (E.-A.B.); 2 Faculty of Electronics, Telecommunications and Information Technologies, Polytechnic University of Timisoara, 1 Mihai Viteazu Ave., 300222 Timisoara, Romania

**Keywords:** dental implants, edentulous jaw, computer-assisted surgery, surgical guides, implant-supported dental prosthesis, screw-retained surgical guide

## Abstract

**Background:** Stabilization of surgical guides in fully edentulous patients remains a clinical challenge due to mucosal resilience and potential micromovement, even when fixation pins are used. Guide instability may affect drilling accuracy and overall workflow predictability. This proof-of-concept case series describes a stabilization approach based on pre-placed tripod reference implants with multi-unit coupling, designed to create a mechanically defined prosthetic docking platform for fully guided implant surgery. **Methods:** Three fully edentulous patients requiring implant-supported rehabilitation were treated using a two-stage protocol. Three temporary reference implants were inserted in a tripod configuration 7–10 days prior to definitive surgery. Multi-unit abutments were mounted on the reference implants, and intraoral scanning was performed to design a surgical guide indexed to the prosthetic interfaces. During implant placement, the guide was screw-retained to the reference implants via the multi-unit connections. Postoperative implant positions were evaluated radiographically by superimposing postoperative datasets onto the preoperative planning model. Intraoperative guide stability, surgical events, and early postoperative outcomes were recorded. **Results:** Stable guide fixation was achieved in all three cases without detectable intraoperative displacement. Implant placement was completed as planned in each patient, and removal of the temporary reference implants was uneventful. No intraoperative or early postoperative complications were observed. Mean coronal, apical, and angular deviations between planned and achieved implant positions were 0.70 ± 0.16 mm, 0.39 ± 0.13 mm, and 3.30 ± 0.59°, respectively. These preliminary findings, derived from four treated arches, were comparable to ranges reported in selected studies on fully guided implant surgery; however, no direct statistical comparison with previously published datasets was performed. **Conclusions:** Within the limitations of this proof-of-concept case series, temporary reference implants arranged in a tripod configuration provided a stable and reproducible prosthetic indexing platform for guided implant surgery in fully edentulous patients. Further prospective studies involving larger patient cohorts and controlled comparative designs with conventional mucosa-supported or fixation-pin-supported surgical guides are required to evaluate the reproducibility, clinical performance, and long-term applicability of this stabilization concept.

## 1. Introduction

Computer-guided implant surgery has become an established component of contemporary implant dentistry, enabling prosthetically driven treatment planning and the translation of virtual implant positioning into the clinical setting through static surgical templates [1,2,3]. Digital workflows combining cone-beam computed tomography (CBCT) and intraoral scanning allow three-dimensional treatment planning based on both anatomical and prosthetic parameters [4,5,6]. The accuracy of this transfer depends not only on imaging quality and software alignment, but also on the mechanical stability and reproducibility of the surgical guide during osteotomy preparation and implant insertion [7,8,9].

In partially dentate patients, tooth-supported guides typically provide stable and reproducible seating due to rigid dental support structures [10]. In fully edentulous patients, however, guide stabilization relies on mucosal support, bone support, fixation pins, or combinations of these methods [11,12,13]. Mucosa-supported templates may be influenced by soft tissue resilience and compressibility, particularly during drilling or under functional loading [14,15]. Fixation pins are commonly used to enhance stabilization, but their placement requires additional surgical steps and does not inherently provide a prosthetically indexed interface or a reproducible mechanical docking system in completely edentulous arches [16,17]. In full-arch guided implant surgery, reproducible guide seating and rotational stability are critical because cumulative positional deviations across multiple implants may directly affect passive prosthetic fit and restorative alignment. In this context, prosthetic indexing may contribute to more reproducible spatial positioning of the surgical guide by establishing a mechanically defined docking interface independent of mucosal compressibility. Bone-supported guides may offer improved stability but typically require flap elevation and may alter the surgical workflow [18]. Consequently, multiple stabilization strategies have been proposed, and no single approach has been universally adopted for all fully edentulous clinical situations.

Multiple technical and clinical factors may influence the accuracy of guided implant surgery, including guide support type, manufacturing tolerances, and intraoperative handling [19,20,21]. In fully edentulous cases, additional mechanical considerations include rotational control and the reproducibility of vertical seating [22,23]. These aspects become particularly relevant in full-arch rehabilitations, where cumulative deviations across multiple implants may influence prosthetic fit and subsequent restorative procedures [24].

Temporary anchorage devices and mini-implants have been widely used in orthodontics for skeletal anchorage and in implant dentistry for provisional stabilization or transitional prosthetic support [25,26,27]. In certain treatment sequences, temporary implants have also been employed to assist provisionalization or staged rehabilitation protocols [28,29]. However, the concept of using pre-placed temporary implants as fixed spatial reference points for the fabrication and stabilization of a screw-retained surgical guide has not been systematically described in the context of guided implant surgery for fully edentulous patients. The present workflow represents a prosthetically indexed modification of existing stabilization principles specifically adapted for completely edentulous arches, in which temporary reference implants serve as digital reference structures and mechanical fixation points for the surgical guide.

The present work describes a technical workflow in which three temporary reference implants are inserted prior to definitive implant placement in fully edentulous patients. Multi-unit and scan abutment components are mounted on these implants to enable CBCT imaging and intraoral digital scanning. The surgical guide is subsequently designed to engage this prosthetic interface in a tripod configuration, creating a screw-retained docking platform that is independent of mucosal compression. After guided placement of the definitive implants, the temporary reference implants are removed during the same surgical session.

The aim of this proof-of-concept case series is to describe the surgical and digital workflow of this stabilization concept and to report intraoperative guide behavior, radiographic deviation between planned and achieved implant positions, and early clinical events in three fully edentulous patients. The present study was designed primarily to evaluate the technical feasibility and preliminary clinical applicability of this prosthetically indexed stabilization workflow in completely edentulous arches rather than to demonstrate superiority over conventional fixation methods. Accordingly, the present work should be interpreted as a technical proof-of-concept description rather than as a comparative efficacy study intended to demonstrate superiority over existing stabilization approaches.

## 2. Materials and Methods

### 2.1. Study Design

This study represents a proof-of-concept technical case series including three fully edentulous patients requiring implant-supported rehabilitation of the maxilla, mandible, or both arches, corresponding to four treated arches in which a total of 23 implants were placed using the described guided surgical protocol. The objective of the study was to document the surgical and digital workflow of a screw-retained surgical guide stabilization concept using temporary reference implants.

All eligible patients who agreed to participate and provided informed consent were included in the study. Each patient underwent comprehensive clinical and radiographic evaluation prior to treatment. Treatment planning included CBCT and digital prosthetic assessment. The indication for guided implant surgery was based on a prosthetically driven rehabilitation concept tailored to the individual clinical situation.

Patients were considered eligible if they presented complete edentulism requiring implant-supported rehabilitation, sufficient bone volume for implant placement without extensive grafting procedures, and the ability to undergo CBCT imaging and guided implant surgery. Additional inclusion criteria included the absence of uncontrolled systemic disease and the possibility of achieving adequate primary stability for placement of the temporary reference implants.

Patients presenting active oral infection, severe uncontrolled systemic conditions contraindicating implant surgery, inability to undergo guided surgery procedures, or insufficient bone availability preventing stable placement of the temporary reference implants were excluded from treatment using the present workflow.

Because the present investigation was designed as a technical proof-of-concept case series intended to evaluate feasibility and workflow applicability, no formal sample size calculation was performed.

The study protocol was conducted in accordance with the Declaration of Helsinki and received approval from the institutional ethics committee (approval number CECS UMFVBT Nr. 114/03.10.2022, revalidated in 2025). Written informed consent was obtained from all patients for surgical treatment and for the use of anonymized clinical data for scientific and publication purposes. In addition, the research aligns with PROCESS checklist that can be found in the Supplementary Material (Table S1).

### 2.2. Temporary Reference Implants and Guide Stabilization Concept

**Figure 1:**
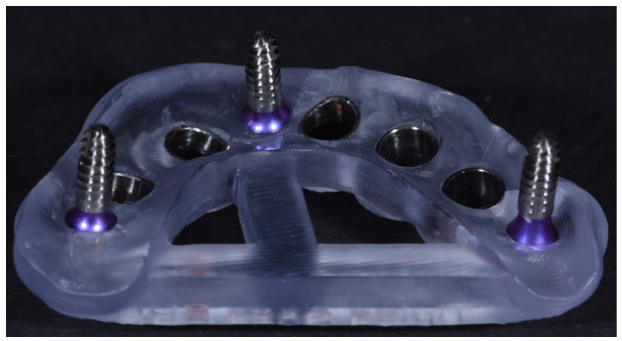
Surgical guide prototype illustrating the tripod fixation concept. Three temporary reference implants engage the guide through multi-unit connections, creating a mechanically defined prosthetic docking interface for guided implant surgery.

The stabilization concept was based on the preoperative placement of three temporary reference implants intended to serve as mechanical anchorage points for the surgical guide (Figure 1). The reference implants consisted of commercially available titanium implants (copaSKY^®^, bredent medical GmbH & Co. KG, Senden, Bavaria, Germany) with dimensions of 3.0 mm × 10 mm. Implant dimensions and geometry were selected to facilitate primary stability suitable for temporary mechanical guide fixation. Implant placement was performed using an under-preparation drilling protocol, with osteotomy preparation limited to pilot drilling in order to maximize primary mechanical stability. Primary stability was clinically measured during insertion using surgical motor torque readings and exceeded 35 Ncm in all temporary reference implants. These implants were removed after final implant placement at the end of the surgical procedure.

The temporary reference implants were inserted freehand using a flapless surgical approach under local anesthesia. Compatible Uni.cone multi-unit abutments (bredent medical GmbH & Co. KG, Germany) were subsequently connected to the temporary implants to establish the prosthetic indexing interface used for guide stabilization. A tripod configuration was adopted to distribute the implants across the arch. The implants were positioned as widely separated as anatomical conditions permitted, typically involving anterior and posterior regions, in order to maximize geometric stability.

The rationale for the tripod configuration is mechanical rather than numerical. Three non-collinear points define a plane and provide intrinsic geometric stability (Figure 1). When distributed across the edentulous arch, this configuration constrains vertical displacement, limits rotational movement, and reduces horizontal translation of the surgical guide. In this arrangement, the reference implants function as fixed anchorage points that define a reproducible spatial relationship between the surgical guide and the underlying bone.

Immediately after insertion, multi-unit abutments were mounted onto the temporary reference implants. Healing screws were initially placed on the multi-unit abutments, and a CBCT scan was obtained with the reference implants and healing screws in situ. The geometry of the healing screws served as a radiographically identifiable reference structure for subsequent alignment with the intraoral scan.

Following CBCT acquisition, the healing screws were replaced with scan abutments to enable accurate capture of the implant interface geometry during intraoral scanning. This sequential workflow allowed reliable transfer of the spatial position of the temporary reference implants into the digital planning environment. The radiographic and optical datasets were subsequently aligned during digital planning in Exocad (Exocad GmbH, Darmstadt, Germany), forming the basis for the design of a screw-retained surgical guide indexed directly to the multi-unit interfaces.

**Figure 2:**
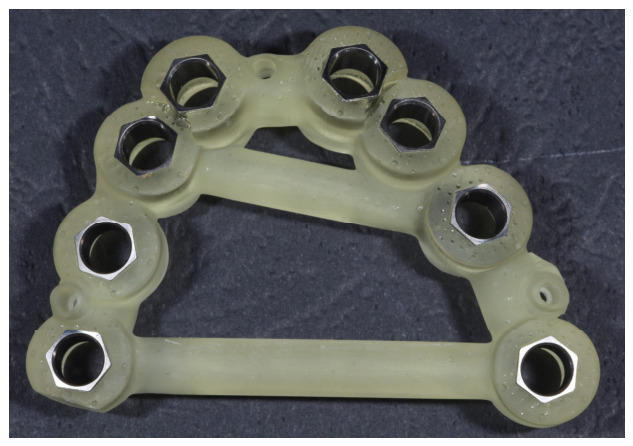
Fabricated sleeve-based surgical guide designed to engage the prosthetic interfaces of the temporary reference implants. The guide incorporates metallic sleeves for drill guidance and internal indexing geometry corresponding to the multi-unit abutments.

The surgical guide was fabricated as a sleeve-based template incorporating internal connections corresponding to the geometry of the multi-unit abutments (Figure 2). Fixation was achieved through screw retention to the reference implants, creating a direct mechanical coupling between the guide and the temporary implants.

This stabilization mechanism differs structurally from mucosa-supported templates. In mucosa-supported guides, seating depends partially on soft tissue adaptation and compression, and stabilization may be supplemented by fixation pins placed after initial positioning. In contrast, the present configuration establishes fixation through direct screw engagement to the reference implants. The final seating position is determined by the prosthetic interface geometry rather than by soft tissue contour. Once secured, the guide becomes mechanically coupled to the reference implants, forming a stable docking interface independent of mucosal resilience.

The reference implants were used exclusively as temporary spatial indexing devices. After guided placement of the definitive implants, they were removed using controlled reverse torque during the same surgical session and were not intended for osseointegration or prosthetic loading.

Within this framework, the temporary reference implants function as spatial indexing devices rather than transitional prosthetic supports. The resulting system enables the fabrication of a screw-retained, sleeve-based surgical guide anchored to a mechanically defined tripod platform in fully edentulous patients.

### 2.3. Workflow

#### 2.3.1. Initial Clinical Assessment and Treatment Planning

**Figure 3:**
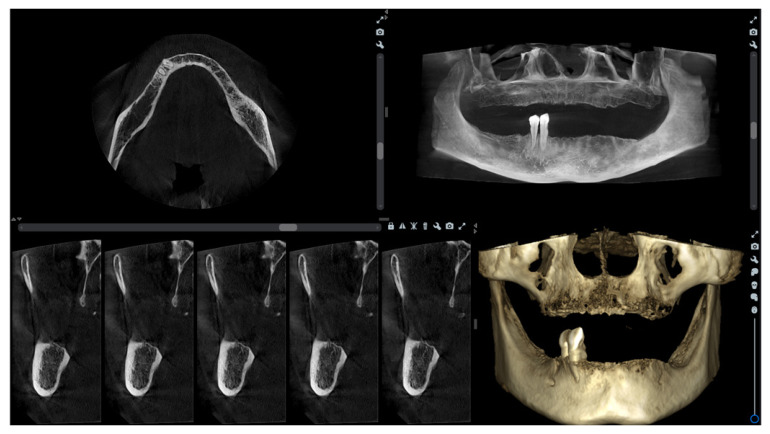
Initial CBCT evaluation of a fully edentulous patient used for treatment planning. Bone volume, anatomical limitations, and prosthetic space were assessed prior to digital implant planning.

Each patient underwent a comprehensive clinical examination and radiographic evaluation during the initial consultation. Bone volume, ridge morphology, and anatomical limitations were assessed using CBCT (Figure 3). Implant therapy was planned as a fully guided procedure based on prosthetically driven principles and the available bone volume.

The definitive implants used in all cases consisted of titanium implants with varying diameters and lengths selected according to ridge morphology, bone availability, and prosthetic space requirements. Implant dimensions were individualized for each clinical situation.

Treatment planning followed a prosthetically driven approach in which the final restoration dictated the optimal implant position, angulation, and distribution within the arch.

#### 2.3.2. Placement of Temporary Reference Implants

In a preliminary surgical phase, three temporary reference implants were inserted freehand in a flapless manner under local anesthesia in a tripod configuration. The implants were distributed across the arch to avoid overlapping with the planned definitive implant positions.

Primary insertion torque exceeded 35 Ncm in all cases, ensuring sufficient mechanical stability for guide fixation.

Multi-unit abutments were mounted immediately after insertion of the temporary reference implants. Healing screws were initially placed on the multi-unit abutments during CBCT acquisition and the first intraoral scan. Scan abutments were subsequently connected during the digital acquisition phase to enable accurate capture of the implant positions.

#### 2.3.3. Radiographic and Digital Data Acquisition

CBCT scans were obtained using a Planmeca ProMax 3D Classic system (Planmeca, Helsinki, Finland) with temporary reference implants and healing screws in situ. A limited field of view was selected according to the treated arch, typically ranging between 5 × 8 cm and 11 × 8 cm. The voxel size was set to approximately 150 μm, and exposure parameters were adjusted according to patient-specific anatomical conditions following standard implant planning protocols.

Following CBCT acquisition, two intraoral scans were performed. The first scan was obtained with the healing screws in place, reproducing the same configuration present during CBCT acquisition. This allowed the geometry of the healing screws to serve as common reference structures between the radiographic and optical datasets. Subsequently, the healing screws were replaced with scan abutments mounted on the multi-unit abutments of the temporary reference implants, and a second intraoral scan was acquired to capture the precise geometry of the implant interfaces.

**Figure 4:**
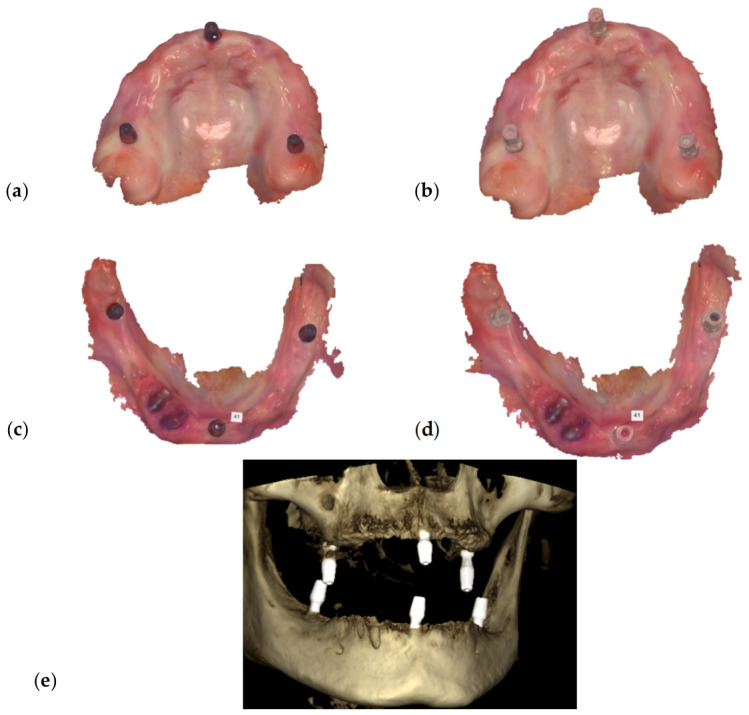
Radiographic and optical datasets used for digital alignment. (**a**) Intraoral scan of the maxillary arch with healing screws mounted on the temporary reference implants. (**b**) Intraoral scan of the maxillary arch after replacement of the healing screws with scan abutments. (**c**) Intraoral scan of the mandibular arch with scan abutments connected to the temporary reference implants. (**d**) Intraoral scan of the mandibular arch with healing screws mounted on the temporary reference implants. (**e**) CBCT reconstruction showing the temporary reference implants used as spatial reference points for alignment of the DICOM and STL datasets.

The intraoral scan containing the healing screws was first aligned with the CBCT dataset using the healing screw geometry as the reference structure visible in both datasets. The intraoral scan containing the scan abutments was then aligned to the first optical scan, thereby transferring the spatial position of the scan abutments into the CBCT coordinate system. This sequential alignment enabled accurate integration of the DICOM and STL datasets for digital implant planning (Figure 4).

#### 2.3.4. Digital Planning and Guide Design

**Figure 5:**
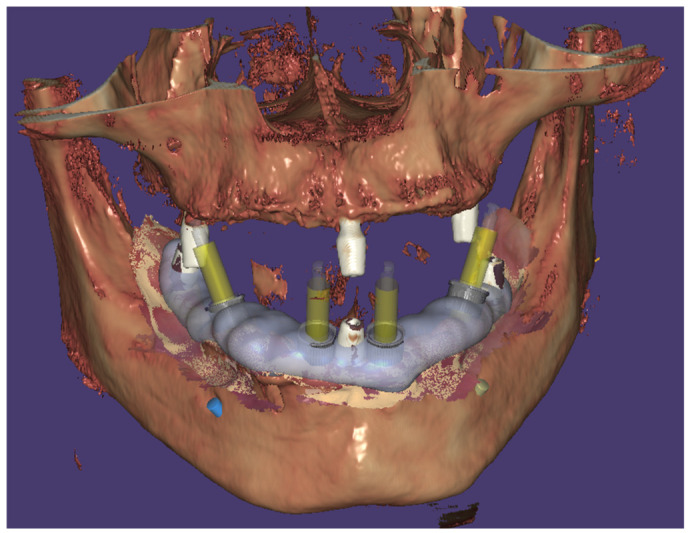
Digital implant planning performed in Exoplan software showing the merged CBCT (DICOM) and intraoral scan (STL) datasets and the planned implant positions within the edentulous arch.

Digital implant planning was performed using exoplan® 3.1 Rijeka software (Exocad GmbH, Darmstadt, Germany). CBCT (DICOM) datasets and intraoral scan (STL) files were imported into the planning software and merged using the spatial relationship previously established between the healing screws and the scan abutments, which served as reference structures for dataset alignment (Figure 5).

Dataset alignment was performed using a combination of automated registration and manual refinement, ensuring precise correspondence between radiographic and optical datasets. Definitive implant dimensions were selected individually during the digital planning phase. Implant diameter and length were determined according to the available bone volume and prosthetic requirements.

Implant positioning followed established surgical and prosthetic principles, including:

- Maintenance of a minimum of 1.5 mm of bone circumferentially around the implant body.
- A minimum inter-implant distance of 3 mm to preserve inter-implant bone peaks and vascular supply.
- Respecting a safety distance of at least 2 mm from critical anatomical structures, including inferior alveolar nerve; mental foramen; maxillary sinus floor; nasal cavity; major vascular bundles when identifiable.

In addition, the following criteria were applied:

- Axial alignment was prosthetically driven to allow favorable screw-retained prosthetic emergence.
- Implant angulation was adjusted to avoid excessive buccal or lingual cortical thinning.
- Primary stability was anticipated based on bone volume and density assessment.
- Avoidance of cortical perforation.
- Preservation of adequate keratinized mucosa when possible.
- Maintenance of restorative space for prosthetic components.

Bone density was assessed qualitatively using CBCT gray-scale distribution and confirmed intraoperatively through tactile feedback during drilling.

All planning decisions were finalized digitally prior to guide fabrication.

#### 2.3.5. Fabrication of the Surgical Guide

The surgical guide was fabricated using an Asiga Max 3D printing system (Asiga, Sydney, Australia) with a biocompatible photopolymer resin suitable for surgical applications. The guide design generated during the digital planning phase was exported as an STL file and prepared for printing according to the manufacturer’s recommended parameters.

Post-processing included removal of support structures, cleaning in isopropyl alcohol, and final polymerization using a dedicated curing unit following the manufacturer’s instructions. Metallic drill sleeves were subsequently inserted into the guide where required for guided implant drilling.

**Figure 6:**
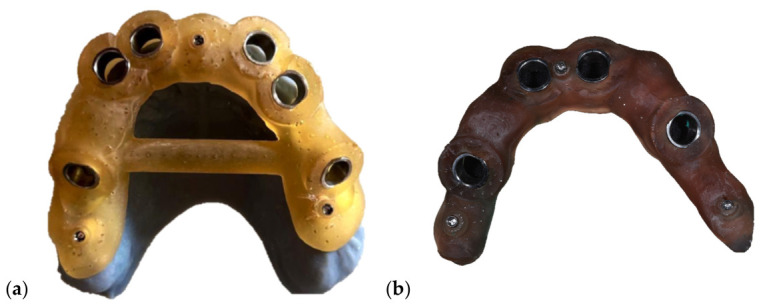
3D-printed surgical guides fabricated following digital implant planning. (**a**) Maxillary surgical guide with inserted metallic drill sleeves. (**b**) Mandibular surgical guide fabricated using the same workflow.

Prior to surgical use, the guide was clinically and laboratory verified on a printed study model according to the standard workflow protocol used in the institution. Guide seating, adaptation, and correspondence with the planned prosthetic workflow were evaluated before sterilization. Subsequently, the guide was sterilized according to the material manufacturer’s recommendations. Two surgical guides can be observed in Figure 6.

### 2.4. Surgery

#### 2.4.1. Preoperative Verification and Surgical Preparation

At the beginning of the definitive implant surgery session, the stability of the temporary reference implants was clinically verified. Each implant was manually tested to confirm maintenance of primary stability, and no mobility was detected in any case.

The multi-unit abutments mounted on the reference implants were inspected to ensure complete seating and absence of soft tissue interference. The surrounding mucosa was evaluated to exclude inflammatory reactions or tissue overgrowth that could interfere with guide positioning.

Local anesthesia was administered according to standard surgical protocol.

#### 2.4.2. Guide Seating and Mechanical Indexing

The sterilized sleeve-based surgical guide was positioned intraorally and aligned with the multi-unit abutments.

Seating was performed in a controlled sequence:

- Initial vertical positioning over the multi-unit interfaces.
- Progressive engagement until complete passive seating was achieved.
- Visual verification of complete contact between guide intaglio surface and prosthetic interfaces.

Because the guide geometry was designed directly on the multi-unit connections, the final seating position was determined primarily by the internal geometry of the guide rather than by mucosal compression.

**Figure 7:**
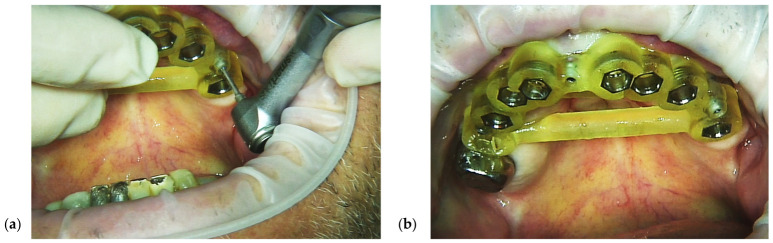
Intraoperative stabilization of the surgical guide using temporary reference implants. (**a**) Fixation of the surgical guide onto the temporary reference implants through the multi-unit interfaces. (**b**) Intraoral view of the fully seated and mechanically stabilized surgical guide prior to the guided osteotomy sequence.

After confirming complete seating, fixation screws were inserted through the integrated screw-access channels and engaged into the multi-unit abutments. Screws were tightened sequentially in a cross-pattern to promote uniform load distribution (Figure 7).

Once tightened, the guide became mechanically coupled with the temporary reference implants.

Mechanical stability was verified by applying controlled manual forces in the vertical, rotational, and horizontal directions. No clinically detectable mobility was observed.

#### 2.4.3. Guided Osteotomy Protocol

Osteotomies were performed using a fully guided drilling protocol compatible with a sleeve-based surgical guide system.

Sequential drilling was carried out through metallic sleeves incorporated within the guide. Each drill was inserted with controlled axial pressure, maintaining continuous irrigation according to standard surgical recommendations.

The sleeve-guided system controlled the following: entry point, angulation, and drilling depth (when depth-controlled drills were used). Bone preparation respected the pre-planned implant dimensions. Throughout the drilling sequence, the guide remained screw-retained to the reference implants, minimizing the risk of intraoperative displacement during osteotomy preparation.

#### 2.4.4. Definitive Implant Insertion

**Figure 8:**
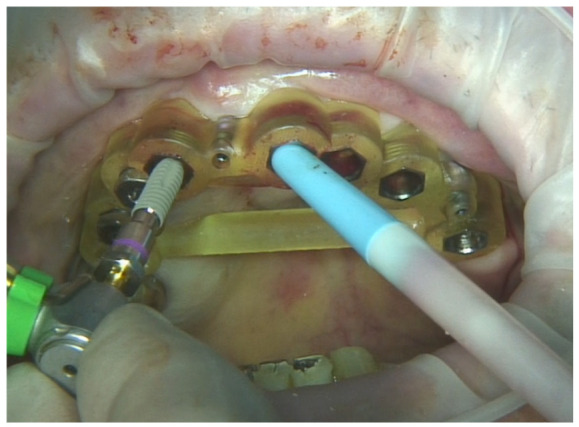
Fully guided implant placement using the implant-stabilized surgical guide.

Definitive implants of variable diameters and lengths were placed according to the preoperative digital plan (Figure 8). Implant dimensions were selected to conform to anatomical availability and prosthetic design constraints as defined during digital planning. Insertion torque was recorded using the surgical motor. In all cases, primary stability exceeded 35 Ncm. The spatial positioning achieved intraoperatively corresponded to the digitally planned implant axes as guided by the screw-retained surgical template.

#### 2.4.5. Removal of Surgical Guide

**Figure 9:**
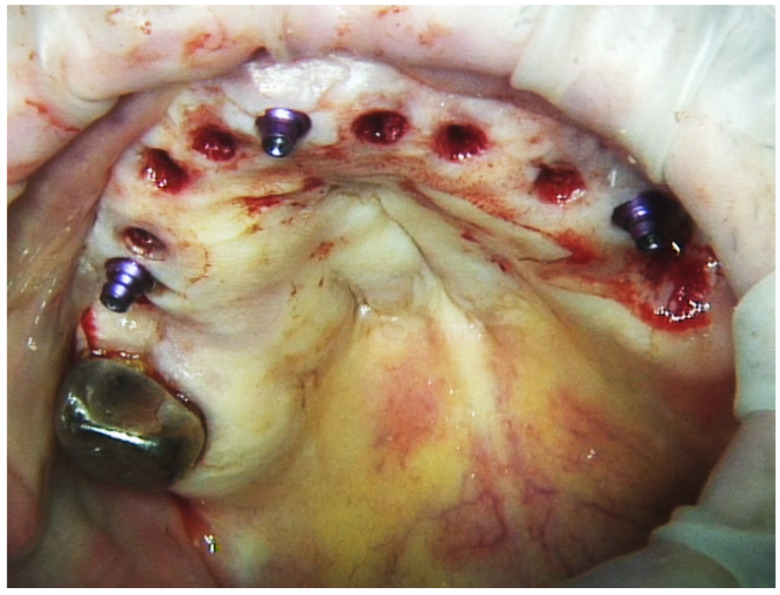
Intraoperative view after removal of the surgical guide following guided implant placement.

After placement of all definitive implants, fixation screws were removed and the surgical guide was detached (Figure 9).

Visual inspection confirmed absence of bone debris accumulation within the guide interface and integrity of the metallic sleeves.

#### 2.4.6. Explantation of Temporary Reference Implants

**Figure 10:**
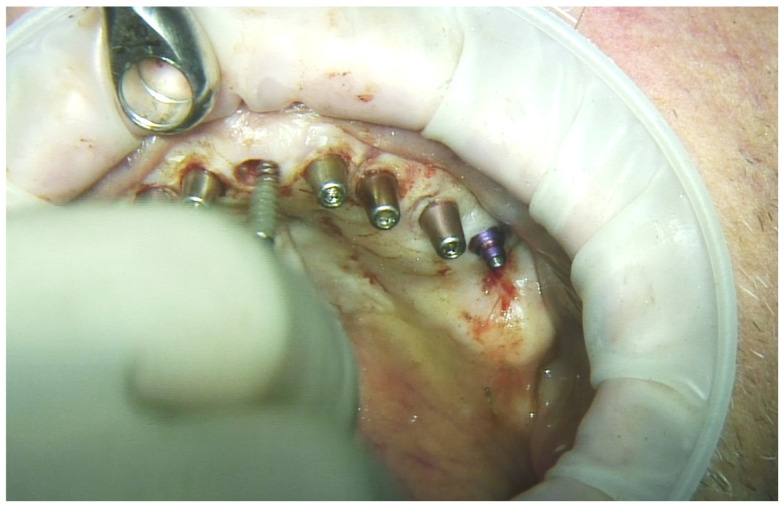
Intraoperative view after removal of one of the reference implants.

Temporary reference implants were removed during the same surgical session (Figure 10). Explantation was performed using controlled reverse torque with a manual torque wrench.

Because these implants were placed for short-term stabilization only, removal required minimal mechanical resistance.

No additional bone preparation was necessary. The explantation sites were inspected for bleeding and spontaneous clot formation following removal of the temporary reference implants. An absorbable collagen sponge was subsequently placed into each explantation socket in order to support clot stabilization and secondary soft tissue healing of the temporary osteotomy sites. Following collagen placement, the surrounding soft tissues were adapted and sutured to promote uneventful postoperative healing.

The reference implants were not intended for osseointegration or prosthetic loading; their role was limited to spatial indexing and intraoperative stabilization.

### 2.5. Outcomes Assessment

Clinical and radiographic outcomes were evaluated following completion of the surgical procedure. Intraoperative guide stability was assessed after fixation of the surgical guide to the temporary reference implants. Mechanical stability was verified by applying controlled manual forces in vertical, rotational, and horizontal directions in order to detect potential guide displacement. Guide seating and stability were monitored throughout the sequential drilling protocol.

Intraoperative and early postoperative events were also recorded for each treated case, including guide instability during drilling, loosening or failure of the temporary reference implants, inability to complete the guided drilling protocol, and surgical complications related to implant placement.

Radiographic accuracy of implant placement was evaluated using cone-beam computed tomography (CBCT) acquired immediately after surgery. The postoperative CBCT datasets were imported into the original Exoplan planning project (Exocad GmbH, Darmstadt, Germany) and superimposed onto the preoperative planning datasets used for surgical guide fabrication.

The initial planning workflow had previously integrated the preoperative CBCT dataset and intraoral scan files using the geometry of the healing screws and scan abutments as common spatial reference structures. Following surgery, the postoperative CBCT was aligned within the same digital planning environment, allowing direct comparison between the planned implant positions and the achieved implant positions visible in the postoperative scan.

For each implant, the following deviation parameters were measured directly within the Exoplan software environment:

- Coronal (platform) deviation—linear distance between the planned and achieved implant platform positions measured in millimeters (mm).
- Apical deviation—linear distance between the planned and achieved implant apex positions measured in millimeters (mm).
- Angular deviation—angular difference between the planned and achieved implant axes measured in degrees (°).

No formal quantitative RMS threshold or external registration validation protocol was established because the present investigation was designed as a technical proof-of-concept case series rather than a formal metrological validation study.

All measurements were performed by a single calibrated examiner (R.-A.E.) using the measurement tools available within the Exoplan software environment (Exocad GmbH, Darmstadt, Germany). Each measurement was repeated three times, and the mean value was recorded for analysis. Repeated measurements demonstrated only minimal variation. The examiner was not formally blinded to the planned implant positions because all measurements were performed within the original digital planning environment used for surgical workflow analysis. No additional statistical reproducibility analysis, such as intraclass correlation coefficient (ICC) calculation, was performed.

Measurements were recorded individually for all 23 implants included in the study.

## 3. Results

### 3.1. Case and Implant Distribution

Three fully edentulous patients corresponding to four treated arches were included in this proof-of-concept case series (Table 1). The treated arches comprised three maxillary rehabilitations and one mandibular rehabilitation. A total of 23 definitive implants were placed using the described guided surgical protocol.

Case 1 involved maxillary rehabilitation with eight implants. Case 2 involved bimaxillary rehabilitation with six maxillary implants and four mandibular implants. Case 3 involved maxillary rehabilitation with five implants.

Each treated arch was stabilized using three temporary reference implants, resulting in a total of 12 temporary reference implants used for surgical guide fixation. The surgical protocol was successfully completed in all treated cases without technical complications.

**Table 1:** Distribution of treated arches and implants included in the study.

Case	Treated Arch	Definitive Implants (*n*)	Temporary Reference Implants (*n*)
1	Maxilla	8	3
2	Maxilla	6	3
2	Mandible	4	3
3	Maxilla	5	3
Total	4 arches	23	12

### 3.2. Intraoperative Guide Stability and Surgical Feasibility

During surgery, all guides demonstrated stable seating and precise mechanical engagement with the temporary reference implants. Once fixation screws were tightened, the guides remained rigidly coupled to the multi-unit interfaces without clinically detectable vertical, rotational, or horizontal displacement.

No guide instability or intraoperative repositioning was required in any of the treated arches. The fully guided drilling protocol was completed as planned in all cases, and all implants were inserted according to the preoperative digital plan.

Because the temporary reference implants provided a stable prosthetic reference interface, a provisional prosthetic framework was temporarily positioned intraoperatively prior to definitive implant placement in order to verify passive fit and spatial correspondence with the planned restoration. Temporary prosthetic components connected to the implants passed through oversized access openings within the provisional restoration. The space surrounding these components was subsequently relined intraorally using a provisional restorative material in order to stabilize and transfer the spatial implant relationship to the provisional framework. Final laboratory refinement and prosthetic finishing were subsequently performed following the surgical procedure.

### 3.3. Radiographic Accuracy of Implant Placement

Radiographic accuracy was assessed by superimposing postoperative CBCT datasets with preoperative digital implant planning. A total of 23 implants placed across four treated arches were included in the analysis.

**Figure 11:**
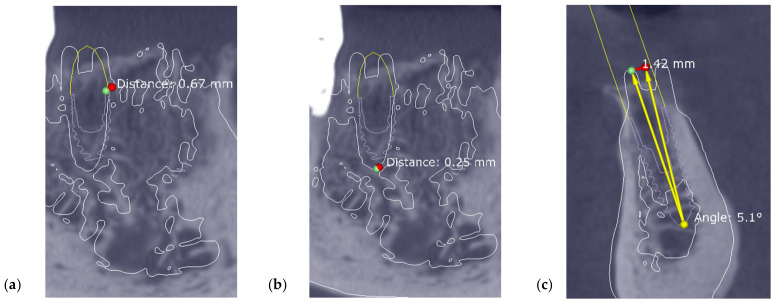
Radiographic accuracy analysis performed by superimposing postoperative CBCT datasets with preoperative implant planning. Representative measurements illustrating coronal deviation (**a**), apical deviation (**b**), angular deviation (**c**).

Three parameters were evaluated for each implant: coronal deviation, apical deviation, and angular deviation between the planned and the achieved implant position (Figure 11).

The mean coronal deviation was 0.70 ± 0.16 mm, with values ranging from 0.47 mm to 1.10 mm.

The mean apical deviation was 0.39 ± 0.13 mm, ranging from 0.23 mm to 0.62 mm.

The mean angular deviation was 3.30 ± 0.59°, with observed values between 2.5° and 5.0°.

These results suggested reproducible transfer of the digital implant planning into the surgical field within the limitations of the present case series. No major positional discrepancies affecting the planned prosthetic workflow were observed intraoperatively.

Mean deviations (Table 2 and Table 3) remained within ranges commonly reported in the literature for fully guided implant surgery.

**Figure 12:**
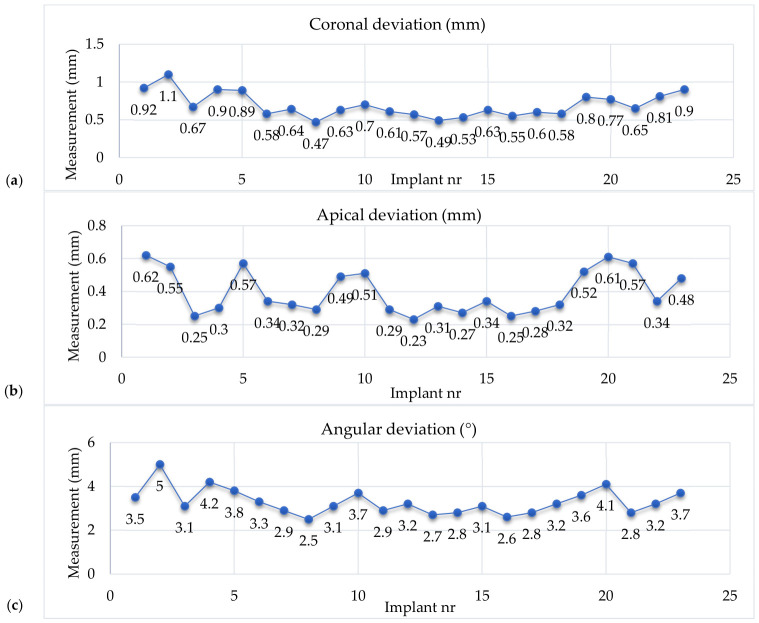
Distribution of implant placement deviations across the analyzed implants. (**a**) Coronal deviation (mm), (**b**) apical deviation (mm), and (**c**) angular deviation (°) measured between planned and achieved implant positions following CBCT superimposition.

To further illustrate the variability of implant placement accuracy across the analyzed implants, the distribution of deviations for each individual implant is presented in Figure 12. The graphical representation allows visualization of the consistency of the guided surgical protocol by showing the coronal, apical, and angular deviations measured for all 23 implants included in the study.

Overall, the deviations remained relatively homogeneous across the implant series, without extreme outlier values. Coronal deviations showed a narrow distribution around the calculated mean values, while apical deviations remained consistently below 0.7 mm for all implants. Angular deviations also demonstrated limited variability, with most measurements clustering around approximately 3°. Although major scattering artifacts were not observed during postoperative evaluation, minor CBCT-related beam-hardening effects and voxel-based interpretation limitations inherent to titanium implant imaging cannot be completely excluded during precise landmark identification.

These findings support the reproducibility of the implant-indexed surgical guide stabilization concept within the limits of the present case series.

## 4. Discussion

a. Article-Related Considerations and Comparison with the Literature

The present proof-of-concept case series evaluated a prosthetically indexed tripod stabilization concept for surgical guides in fully edentulous patients and showed preliminary deviations that were comparable to those reported in selected guided implant surgery studies. The mean coronal deviation observed in this study was 0.70 ± 0.16 mm, the mean apical deviation was 0.39 ± 0.13 mm, and the mean angular deviation was 3.30 ± 0.59°. These values were comparable to those reported in selected studies on static computer-guided implant surgery; however, direct comparisons should be interpreted cautiously because of differences in guide design, support type, registration workflow, and measurement methodology [30].

The relatively low mean apical deviation observed in the present study should be interpreted cautiously. Differences in radiographic superimposition methodology, dataset registration workflow, software environment, and measurement protocols may significantly influence the reported deviation values between studies. In the present workflow, postoperative CBCT datasets were aligned within the original Exoplan planning project used for surgical guide fabrication, allowing comparison between planned and achieved implant positions within the same digital coordinate system. This methodology may differ from alternative protocols using external metrological software or best-fit alignment approaches based on non-implant anatomical structures. In addition, the use of mechanically indexed temporary reference implants positioned within the same treated arch may have contributed to stable guide positioning and reduced spatial displacement during osteotomy preparation. However, this observation should be interpreted as a preliminary technical hypothesis rather than evidence of superior accuracy and requires validation through controlled comparative studies involving larger patient cohorts. In addition, the reported deviation values should be interpreted as workflow-specific descriptive measurements obtained within the native planning environment and not as independently validated metrological accuracy outcomes.

Another possible explanation for the relatively low apical deviation may relate to the anatomical constraints of the treated edentulous arches. In several cases, the available bone volume limited the range of possible implant apex displacement, meaning that minor angular deviations tended to produce proportionally greater coronal displacement while the implant apex remained confined within the available osseous envelope. This biomechanical relationship may partially explain the discrepancy between coronal and apical deviation values observed in the present series.

Previous articles have reported mean coronal deviations typically ranging from approximately 0.9 to 1.4 mm, apical deviations between 1.2 and 1.6 mm, and angular deviations between 3° and 5° [31]. Studies specifically evaluating pin-supported surgical guides in fully edentulous models have reported comparable ranges of deviation, generally between approximately 0.8 and 1.3 mm coronally, 1.0 and 1.6 mm apically, and 3° to 5° angularly, depending on fixation protocol and surgical conditions [12,13,22]. Although comparisons between studies should be interpreted cautiously due to differences in guide support type, registration workflows, software environments, and measurement methodologies, the observed deviations should be interpreted exclusively as preliminary findings supporting the technical feasibility of the proposed workflow. They do not demonstrate superiority over conventional fixation-pin, mucosa-supported, bone-supported, or stackable-guide protocols. The relatively low apical deviations observed in the present series may be partially influenced by the rigid prosthetically indexed fixation provided by the temporary reference implants and by the use of a shared digital planning environment for postoperative superimposition. However, because the present study did not include a comparative control group or an independent registration validation protocol, these findings should be interpreted cautiously.

A key conceptual difference of the proposed workflow is the use of a prosthetically indexed stabilization system instead of a conventional mucosa-supported reference. In mucosa-supported guides, seating may be influenced by soft tissue compressibility, whereas the tripod stabilization concept relies on rigid prosthetic interfaces to create a mechanically defined docking platform largely independent of mucosal resilience [32]. Because fixation is achieved through direct screw retention to temporary reference implants, guide stability is maintained even when surgical flap elevation is performed. This characteristic allows the clinician to perform open-flap procedures when indicated without compromising guide positioning accuracy, which may represent a practical advantage in cases requiring bone reduction, regenerative procedures, or direct visualization of anatomical structures. However, the present study was not designed to directly compare the proposed workflow with fixation-pin-supported, mucosa-supported, bone-supported, or stackable-guide systems, and therefore no conclusions regarding comparative performance can be drawn.

Furthermore, the mechanical fixation provided by the reference implants permits the intentional design of a controlled vertical offset between the guide and the mucosal surface. In clinical practice, maintaining a clearance of approximately 2 mm between the guide and the soft tissues may facilitate irrigation, surgical visibility, and access during flap procedures while preserving guide stability through screw fixation.

An additional clinical consideration relates to the presence and quality of keratinized mucosa. When keratinized tissue is insufficient, a flapless approach may not be appropriate due to the increased risk of soft tissue complications and compromised peri-implant tissue stability. In such situations, flap elevation may be indicated to allow proper soft tissue management. The stability of the screw-retained tripod fixation system enables the guided surgical workflow to be maintained under these conditions.

Although the proposed workflow offers a prosthetically indexed fixation interface, it also introduces additional surgical and prosthetic steps compared with conventional mucosa-supported guided surgery. These include the preliminary placement of temporary reference implants, the connection of multi-unit and scan components, additional digital acquisition steps, and subsequent removal of the reference implants. Therefore, the rationale of the workflow is not to reduce procedural complexity, but to provide a mechanically indexed docking interface in fully edentulous arches, where conventional guide seating may be affected by mucosal compressibility and the absence of rigid dental support. Although fixation-pin-supported guides remain biologically less invasive and are widely validated clinically, the present concept was developed in response to stabilization challenges occasionally encountered in selected edentulous cases, where reproducible rotational and vertical seating stability remained clinically difficult to achieve. Fixation-pin protocols also require auxiliary surgical intervention, but they do not inherently provide the same prosthetic indexing interface.

From a biomechanical perspective, three non-collinear points define a stable plane, limiting rotational, vertical, and horizontal movement of the surgical guide. This geometric stability may explain the consistent intraoperative behavior observed in the present case series.

Contemporary stackable or multi-piece guided surgery systems represent one of the most advanced stabilization approaches for complex fully edentulous rehabilitations, particularly in immediate loading and bone reduction protocols. Compared with these workflows, the present prosthetically indexed tripod concept was not intended as a replacement strategy, but rather as a simplified stabilization alternative for selected edentulous cases requiring reproducible guide seating and mechanical fixation. While stackable systems rely on multiple sequential guide components and fixation stages, the proposed workflow uses three temporary prosthetically indexed reference implants to establish a stable tripod fixation geometry within the treated arch. The proposed workflow offers an alternative stabilization strategy based on prosthetic indexing and mechanical fixation within the treated arch. Therefore, no conclusions regarding comparative superiority can be drawn from the present proof-of-concept case series.

b. Provisional Prosthetic Verification

Beyond surgical guide stabilization, an additional practical advantage of the proposed workflow is the possibility of verifying the provisional prosthetic restoration prior to definitive implant placement. Because the temporary reference implants function as fixed spatial reference points within the digital planning workflow, a provisional prosthetic framework can be fabricated digitally and connected intraoperatively to the reference implants before the guided drilling sequence begins.

This step allows clinical evaluation of prosthetic parameters such as passive fit, emergence profile, occlusal relationships, and spatial correspondence with the planned restoration. In the present workflow, the provisional restoration was used exclusively as a verification tool rather than as an immediate loading strategy. The primary objective remained the stabilization of the surgical guide and the accurate transfer of the digital treatment plan.

**Figure 13:**
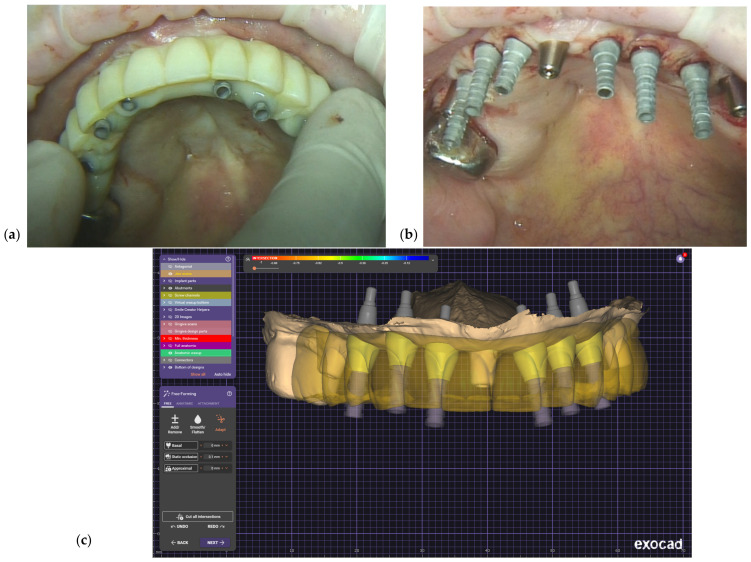
Intraoperative prosthetic verification enabled by the tripod stabilization concept. (**a**) Provisional prosthetic restoration positioned intraoperatively on the temporary reference implants prior to definitive implant placement. (**b**) Clinical view of the definitive implants following guided placement. (**c**) Digital prosthetic design in Exocad showing the relationship between the planned implant positions and the provisional restoration.

In conventional guided surgery workflows, provisional restorations are typically evaluated only after implant placement, when deviations between planned and achieved implant positions may already influence prosthetic outcomes [33]. In contrast, the tripod stabilization concept enables a form of pre-implant prosthetic verification, allowing the clinician to confirm the compatibility between the planned prosthetic design and the surgical reference system before definitive implant insertion (Figure 13). This capability may provide an additional intraoperative verification step during full-arch rehabilitation.

c. Potential for Reduced Procedural Costs

In full-arch implant rehabilitation, the need for multiple fixation points may increase the cost of guided surgery, particularly when single-use components are required [34,35]. From a practical perspective, the present workflow currently requires the use of three temporary reference implants for each treated arch, which may represent an additional procedural cost during guided full-arch rehabilitation. However, if temporary reference implants specifically designed for short-term mechanical stabilization were developed and validated for repeated sterilization according to regulatory standards and manufacturer recommendations, the possibility of controlled reuse could potentially reduce the cost associated with this stabilization workflow.

The present study did not evaluate implant reuse, sterilization protocols, or cost-effectiveness. Therefore, no economic conclusions can be drawn from the current data. Future research may investigate whether dedicated temporary reference implants specifically designed for short-term stabilization could be developed and validated for this application.

d. Limitations and Future Work

The present study has several limitations that should be considered when interpreting the results. First, the study design represents a technical proof-of-concept case series with a limited number of patients and treated arches. Second, the absence of a control group prevents direct comparison with conventional mucosa-supported or bone-supported surgical guides. Third, the evaluation focused primarily on short-term surgical outcomes and radiographic implant positioning, while long-term prosthetic and biological outcomes were not assessed. In addition, standardized long-term postoperative evaluation of peri-implant soft tissue healing at the temporary implant explantation sites and long-term monitoring of the definitive implants were not available for all treated cases at the time of manuscript preparation. In addition, no formal blinding protocol, independent third-party metrological validation, quantitative registration validation, intra-examiner reliability assessment, or inter-examiner reproducibility testing was performed. The use of the native Exoplan planning environment for postoperative deviation assessment was intended to preserve the original virtual implant positions used for surgical guide fabrication and to reduce variability related to inter-software conversion, coordinate transformation, implant library differences, and inter-observer landmark selection. However, this approach does not represent independent third-party metrological validation, and the reported deviation values should therefore be interpreted as workflow-specific descriptive measurements. Future studies should also incorporate formal intra-examiner and inter-examiner reliability assessment to further evaluate measurement reproducibility.

Several potential technical and biological limitations of the proposed workflow should also be considered. Because guide stabilization depends on the mechanical integrity of the temporary reference implants, loss of implant stability between the preliminary and definitive surgical phases could compromise guide fixation and require modification of the surgical workflow. Although no loss of stability occurred in the present case series, this possibility represents a potential limitation of the technique.

In the present workflow, the temporary reference implants were strategically positioned outside the planned definitive implant configuration in order to avoid mechanical interference during osteotomy preparation and implant insertion. Typically, the anterior reference implant was placed in central incisor regions where definitive implants were not planned, while the posterior reference implants were positioned distally relative to the definitive implant distribution, often in molar regions. This spatial distribution allowed creation of a stable tripod configuration while preserving adequate safety distance from the planned definitive implant trajectories. However, anatomically restricted arches with limited posterior bone volume may still represent challenging situations requiring careful digital planning.

Furthermore, although no biological complications were observed during the 7–10-day healing period, temporary transmucosal implants may theoretically increase the risk of plaque accumulation, soft tissue irritation, or local contamination if appropriate hygiene and peri-implant soft tissue management are not maintained.

The biological consequences of temporary reference implant placement and removal should also be interpreted cautiously. Although no clinically evident cortical fractures, soft tissue dehiscence, healing disturbances, or implant-site complications were observed in the present case series, the creation of temporary osteotomy sites inherently represents additional surgical intervention compared with conventional fixation-pin protocols. Consequently, future studies incorporating standardized radiographic follow-up and larger patient cohorts will be necessary to evaluate potential bone remodeling and long-term tissue response at the temporary implant sites.

The reported implant deviation measurements were obtained within the native Exoplan planning environment used for guide design and surgical execution. Although this approach preserved consistency between the original treatment plan and postoperative evaluation, independent third-party metrological validation was not performed. Consequently, the reported values should be interpreted as workflow-specific descriptive measurements rather than independently validated metrological accuracy outcomes.

Future prospective studies involving larger patient cohorts, controlled comparative designs, independent metrological validation protocols, examiner reproducibility assessment, and longer follow-up periods will be required to formally validate the clinical performance of the proposed concept. Additional research may also investigate long-term implant survival, prosthetic complications, biological outcomes, patient-centered outcomes, and the mechanical repeatability of the prosthetically indexed fixation interface under different clinical conditions.

## 5. Conclusions

This proof-of-concept case series demonstrates that the use of temporary reference implants arranged in a tripod configuration can provide a stable and reproducible prosthetic indexing system for surgical guide positioning in fully edentulous patients. The observed deviations between planned and achieved implant positions were comparable to those reported in selected studies on static computer-guided implant surgery. However, due to the proof-of-concept design, limited sample size, and absence of a comparative control group, these findings should be interpreted cautiously.

Beyond surgical accuracy, the described workflow enables intraoperative verification of the provisional prosthetic restoration prior to definitive implant placement. This capability may serve as an additional clinical safety mechanism, allowing clinicians to confirm prosthetic alignment and spatial relationships before completing the guided implant procedure and potentially improving the predictability of full-arch implant rehabilitation.

While the present findings are encouraging, further prospective studies with larger patient cohorts and comparative designs are required to validate the clinical performance and long-term outcomes of this approach and to define its role within contemporary guided implant surgery workflows.

## Figures and Tables

**Table 2:** Radiographic deviation between planned and achieved implant positions.

Parameter	Mean ± SD	Minimum	Maximum
Coronal deviation (mm)	0.70 ± 0.16	0.47	1.10
Apical deviation (mm)	0.39 ± 0.13	0.23	0.62
Angular deviation (°)	3.30 ± 0.59	2.5	5.0

**Table 3:** Implant distribution and deviation values per case.

Case	Arch	Implants (*n*)	Mean Coronal Deviation (mm)	Mean Apical Deviation (mm)	Mean Angular Deviation (°)
Case 1	Maxilla	8	0.75	0.42	3.51
Case 2	Maxilla and Mandible	10	0.57	0.28	2.91
Case 3	Maxilla	5	0.78	0.50	3.48

## Data Availability

The data supporting the findings of this study are not publicly available due to privacy and ethical restrictions but are available from the corresponding author upon reasonable request. Clinical videos documenting the surgical procedures performed in this study are not publicly available due to patient privacy and institutional restrictions but are available from the corresponding author upon reasonable request. Additional technical documentation related to the implant systems and auxiliary materials used in the study (e.g., manufacturer brochures and technical specifications) is also available upon request.

## Supplementary Materials

The following supporting information can be downloaded at https://www.mdpi.com/article/10.3390/dj14060385/s1, Table S1: PROCESS 2023 checklist and location of each reporting item within the manuscript.

## Abbreviations

The following abbreviations are used in this manuscript:

CBCT	Cone-Beam Computed Tomography
3D	Three-Dimensional
CT	Computed Tomography
STL	Standard Tessellation Language
FOV	Field of View
SD	Standard Deviation
