# Supplementary material for: A Prosthetically Coupled Tripod Fixation Concept for Edentulous Surgical Guides: A Three-Case Proof-of-Concept Study
Source: Dent J (Basel). 2026 Jun 22;14(6):385. doi: 10.3390/dj14060385 (PMC13298028; doi:10.3390/dj14060385)
Supplement: Supplementary file 1 [file dentistry-14-00385-s001.zip › dentistry-4258971-supplementary.pdf]

PROCESS 2023 Item	Description	Location in Manuscript
1	Title identifies the study as a proof-of-concept case series	Title page
2	Structured abstract summarizing background, methods, results, and conclusions	Abstract
3	Scientific background and rationale	Introduction
4	Objectives / purpose of the study	End of Introduction
5	Study design	Section 2.1 – Study Design
6	Patient eligibility and recruitment	Section 2.1 – Study Design
7	Ethical approval and informed consent	Section 2.1 – Study Design
8	Clinical setting and workflow description	Sections 2.2–2.4
9	Description of surgical technique/intervention	Sections 2.2–2.4
10	Device/material identification	Sections 2.2–2.5
11	Digital workflow and imaging methodology	Sections 2.3.3–2.3.4
12	Outcome assessment methods	Section 2.5 – Outcomes Assessment
13	Radiographic accuracy measurements	Section 2.5 + Section 3.3
14	Results and clinical outcomes	Section 3 – Results
15	Adverse events or complications	Sections 3.2–3.3
16	Limitations of the study	Section 4.d – Limitations and Future Work
17	Interpretation of findings	Discussion
18	Generalizability and future research	Discussion + Conclusions
19	Conclusions supported by findings	Section 5 – Conclusions
20	Conflicts of interest and funding	End sections
21	Data availability statement	End sections
